# Effectiveness of adherence to a renal health program in a health network in Peru

**DOI:** 10.11606/s1518-8787.2020054002109

**Published:** 2020-08-10

**Authors:** Jessica Bravo-Zúñiga, Enrique M. Saldarriaga, Ricardo Chávez-Gómez, Jungmei Gálvez-Inga, Renzo Valdivia-Vega, Mirko Villavicencio-Carranza, José Espejo-Sotelo, Carola Medina-Sal y Rosas, Víctor Suarez-Moreno, Yamilee Hurtado-Roca

**Affiliations:** I Departamento de Nefrología Hospital Nacional Edgardo Rebagliati Martins EsSalud Lima Perú Unidad de Salud Renal, Departamento de Nefrología, Hospital Nacional Edgardo Rebagliati Martins, EsSalud. Lima, Perú; II Instituto de Evaluación de Tecnologías en Salud e Investigación EsSalud Lima Perú Instituto de Evaluación de Tecnologías en Salud e Investigación (IETSI), EsSalud. Lima, Perú; III CHOICE SeattleWashington USA Policy and Economics (CHOICE) Institute. The Comparative Health Outcomes. Seattle, Washington, USA; IV Sin Brechas S.A.C Perú Sin Brechas S.A.C.

**Keywords:** Chronic Renal Insufficiency, prevention & control, Delivery of Health Care, Integrated, Interdisciplinary Communication, Evaluation of the Efficacy-Effectiveness of Interventions

## Abstract

**OBJECTIVE:**

To evaluate the effectiveness of adherence to a multidisciplinary renal health program in reducing mortality and progression to hemodialysis.

**METHODS:**

We used a database that included patient monitoring (2013-2017), dialysis admissions and all cause of mortality in Peru. Adherence to the program was established by meeting minimum visits during the first year of monitoring. The outcome of interest was hemodialysis admissions or all cause-mortality. Kaplan-Meier curves, Log-Rank test and competing survival analysis methods were used to estimate the differential risk between adherent and non-adherent patients.

**RESULTS:**

A total of 20,354 participants was evaluated; 54.1% were male, 72.1 years old in average, 2.2 years average follow-up, and 15,279 (75.1%) belonged to the early stages (1 to 3a) of Chronic Kidney Disease. Adherence decreased the risk of renal replacement therapy in 41.0% (HR = 0.59, 95%CI 0.41–0.85) in the low-risk group and mortality in the high-risk group was 31.0% (HR = 0.69, 95%CI 0.57–0.83).

**CONCLUSIONS:**

The multidisciplinary care strategy with standardized assessments by stage is effective in reducing admission to .0when the patient is identified in early stages and in reducing mortality in advanced stages.

## INTRODUCTION

The overall prevalence of chronic kidney disease (CKD) for all life stages has been estimated at between 11.0 and 13.0%. The prevalence of CKD in Peru is 13.2%^1^. However, in Lima, the country’s capital and where a third of the population resides, it has been estimated at 20.7%^2^. Kidney disease is the sixth leading cause of death in the country, with a 28.0% increase in the last 10 years. The cities that show the greatest growth in the proportion of deaths from CKD are those located in the Sierra region, among the most important reasons are the limited number of nephrologists^3^, and the subsequent delay in receiving care from a specialist^4^.

The natural progression of CKD leads patients in its final stages to the use of renal replacement therapy (RRT). The number of patients on peritoneal dialysis or hemodialysis is 415 patients per million in habitants (pmi) in Peru^5^. Although this is lower than the average in Latin America (660 pmi)^6^. The total of 50.0% of patients with end-stage CKD do not have access to RRT. The Peruvian Ministry of Health recognized that demand has far exceeded national supply^5^.

The economic impact of CKD is global, reaching all countries, regardless of the level of development and health model. While the annual spending on RRT can reach 100,000 USD, the investment in preventive measures to slow the CKD averages 10,000 USD. Hence the importance of primary and secondary prevention of CKD as a health care policy^7^.

Only 30% of Peruvians have social security, and it is in this health system where dialysis generates a significant financial burden. In 2017, expenditure on dialysis exceeded 1.2 million soles (around 363,000 USD), equivalent to 6% of the institutional budget^8^. Additionally, undergoing RRT has a significant negative impact on the quality of life of patients. Reducing the number of new patients requiring RRT is a highly desirable scenario at the individual and social levels.

CKD management strategies are required to delay progression to dialysis or death. According to evidence, the most successful interventions include multidisciplinary care, control of the underlying disease and drug treatment to control modifiable risk factors. Follow-up and adherence are the key aspects for its success^9^.

Non-adherence to chronic drug therapy significantly increases the disease burden. Factors associated with poor adherence include: drugs’ cost, treatment complexity and adverse effects, inadequate monitoring, weak patient-physician relationships and barriers to accessing health care facilities, which are more common in developing economies^10^.

The World Health Organization (WHO) promotes tactics aimed at timely management of disease and associated comorbidities, such as diabetes and high blood pressure (HBP)^11^. Embedded in this context and considering the recommendations of the WHO, the Renal Health Unit of the E. Rebagliati National Hospital, from Peru’s Social Security – EsSalud began the implementation of a secondary prevention plan in 2013. This intervention focuses on multidisciplinary management (including physician, nurse, nutritionist) of patients with risk factors for CKD such as diabetes, hypertension and age over 55, with the aim of reducing the progression of CKD, controlling underlying diseases and reducing overall mortality^12^. The aim of this study was to evaluate the effectiveness of adherence to a renal health program (RHP) in reducing mortality and progression to hemodialysis.

## METHODS

Analysis of monitoring data from a cohort of patients enrolled in the renal health program (RHP) of the E. Rebagliati Hospital and its healthcare network that includes primary care facilities, Lima-Peru, from January 1, 2013 to December 31, 2017. The monitoring ended on July 31, 2018.

Three data bases were used: 1) Follow-up of the patients included in the RHP between 2013 and 2017; 2) Admissions to hemodialysis in the Rebagliati Network, which included starting dates of treatment; and 3) Overall mortality, constructed from the data (until July 2018) of the national registry of identification and civil status of Peru. The three files were provided with a unique alphanumeric code of identification (CI) for each patient, ensuring the anonymity of the information. With these three databases, a unique CI was designed including 23,144 subjects. We excluded data from subjects without a CKD diagnosis (n = 1,429), in stage 5 CKD (n = 393), because they were already receiving dialysis treatment, under 18 years old (n = 19), outside the filtering period (n = 5), inconsistent data (n = 125), missing data (without CKD stage = 424 and date of death = 542).

The exposure variable showed adherence to the RHP. An adherence threshold based on compliance with visits during the first year of admission was established by experts from the Renal Health Unit consultation: for patients with stage 1 and 2 CKD = minimum one visit, for stage 3a and 3b CKD = minimum two visits and for stage 4 CKD = minimum three visits. The stages of CKD were categorized according to their risk of progression (considering GFR and albuminuria) as CKD with low risk of progression: Stage 1 (estimated glomerular filtration rate [eGFR] > 90 ml/min and albumin creatinine ratio [ACR] > 30mg/g), Stage 2 (eGFR 90 < TFGe < 60 ml/ min and ACR > 30mg/g), and Stage 3a (60 < eGFR < 45 ml/min and ACR < 300mg/g); and CKD with high risk of progression: Stage 3a (60 < eGFR < 45 ml/min and ACR > 300mg/g), Stage 3b (45 < eGFR < 30ml/min), and Stage 4 (15 < eGFR < 30ml/min), according to KDIGO guidelines^13^. Risk factors included hypertension, diabetes mellitus and others (including: renal lithiasis, primary and secondary glomerulopathies, obstructive uropathies, and age over 55 years).

The outcome of the analysis was defined by time to hemodialysis or death (from admission until the presentation of either event, whichever occurred first). The effectiveness of adherence to the program was measured in terms of the presence of these outcomes.

For the survival analysis, Kaplan-Meier curves were used and a difference in distribution test (Log-Rank test) was applied to determine whether the curves were statistically different from each other. Survival models were used to evaluate the association between RHP adherence and the presence of hemodialysis or death. This association was crudely estimated and adjusted for the covariates identified as potential confounders of the main association (gender, age categorized in tertiles, risk factors and CKD stages). In the case of progression to hemodialysis, a competitive risk survival analysis was performed by calculating, from parametric regression, specific cumulative incidence curves for each risk^14^. The “Cause-specific competing-risk survival analysis” package RStudio was used for the estimation^15^. Cox regressions were used for proportional Hazards in the case of death as an event. In both cases, Hazard ratios (instantaneous risk) were obtained, with a 95% confidence level and p < 0.05. The analyses were performed using the statistical package RStudio 3.5.0^16^.

This study was approved by the Research and Institutional Ethics Committee of the Edgardo Rebagliati Martins Hospital.

## RESULTS

The total of 20,354 participants were evaluated. Of these, 54.3% were male and had an average age of 72.9 years [standard deviation (SD) = 12.6], mostly with stage 3a CKD (41.4%) and with high blood pressure as the most frequent risk factor (38.7%). The average monitoring time was 2.2 years (SD = 1.4), with 1.7% cases of hemodialysis starting and 10.9% deaths because of all causes during the monitoring period. Of the total population evaluated, 23.6% met the criteria to be considered adherent to the renal health program and 75.1% were identified in the low-progression risk group (Table 1).

**Table 1:** Characteristics of patients in the renal health program (RHP), according to adherence, Health network Rebagliati-Lima Peru, 2013–2017.

Characteristic	Total		Adherence to RHP		No adherence to RHP	
	n	%	n	%	n	%
	20,354		4,801	23.6	15,553	76.4
Monitoring time (years)*	2.2 (1.4)		2.4 (1.3)		2.1 (1.4)	
Admission age (years)*	72.9 (12.6)		72.6 (12.3)		73.1 (12.7)	
Age in tertiles						
Tertile 1 [18–68]	6,514	32.0	1,558	32.5	4,956	31.9
Tertile 2 [69-79]	6,993	34.4	1,747	36.4	5,246	33.7
Tertile 3 [80-103]	6,847	33.6	1,496	31.2	5,351	34.4
Initial CKD stage						
1	3,614	17.8	1,053	21.9	2,561	16.5
2	4,999	24.6	1,683	35.1	3,316	21.3
3a	8,433	41.4	1,376	28.7	7,057	45.4
3b	2,207	10.8	531	11.1	1,676	10.8
4	1,101	5.4	158	3.3	943	6.1
Initial CKD stage categorized						
Low-risk CKD	15,279	75.1	3,687	76.8	11,592	74.5
High-risk CKD	3,708	18.2	795	16.6	2,913	18.7
Gender						
Women	9,294	45.7	2,438	50.8	6,856	44.1
Men	11,06	54.3	2,363	49.2	8,697	55.9
Risk factors						
Others	5,347	26.3	1,012	21.1	4,335	27.9
Hypertension	7,877	38.7	1,763	36.7	6,114	39.3
Diabetes	2,795	13.7	765	15.9	2,03	13.1
Diabetes and HBP	4,335	21.3	1,261	26.3	3,074	19.8
Total events (dialysis start)	347	1.7	92	1.9	255	1.6
Total of deaths	2,224	10.9	491	10.2	1,733	11.1

* Average (standard deviation)

CKD – chronic kidney disease; HBP: high blood pressure

**Figure 1:**
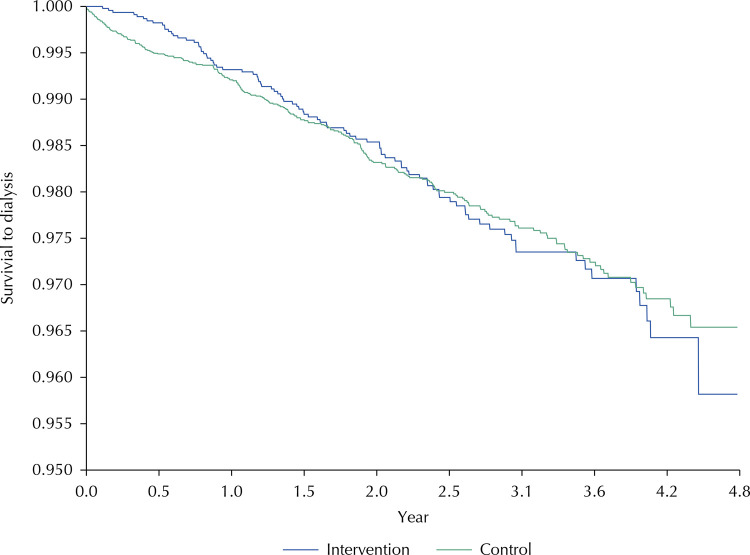
Kaplan-Meier curves to evaluate progression to hemodialysis in patients of the renal health program according to adherence, Health network Rebagliati-Lima Peru, 2013–2017.

The Kaplan-Meier curves for both groups with the beginning of dialysis as the event of interest showed a proportion of people not on dialysis exceeding 95% at the end of five years of monitoring (Figure 1). These proportions were consistent with the small number of people who go on RRT (Table 1). Dialysis survival was lower in the RHP adherent group (95.8%; 95%CI 94.3–97.3) than in the control (96.5; 95%CI 95.9–97.1). However, when applying the Long-Rank test it was evident that the curves were not significantly different from each other (p-value = 0.093); therefore, adherence to the RHP did not modify admission to RRT in the overall sample.

**Figure 2:**
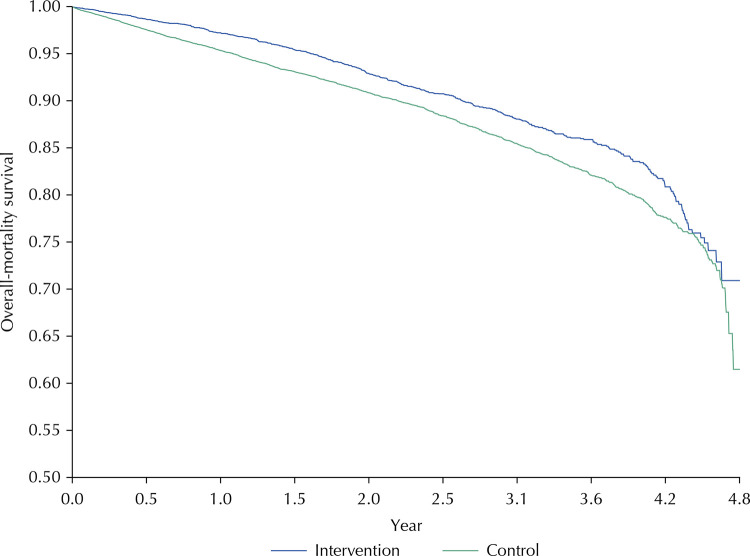
Kaplan-Meier curve to evaluate mortality in patients of the renal health program, according to adherence, Health network Rebagliati-Lima Peru, 2013–2017.

In the Kaplan-Meier overall mortality curves for both groups, the group that did not adhere to the RHP had a lower survival than the adhered group throughout the study period (Figure 2). At the end of the following five years, survival was 70.9% (95%CI 65.8–76.8) in the adherent group and 61.5% (95%CI 55.1–68.6) in the non-adherent group. The Long-Rank test showed that the curves were statistically different from each other (p < 0.001), so adherence to the RHP would modify mortality.

To evaluate the effect of adherence to the RHP on the start of hemodialysis, crude and adjusted regression analyses were performed, showing a change in the directionality of the association. Hence the stage of CKD would be an effect modifier on this association (data not shown). Thus, the results indicated that adherence to the RHP had differentiated effects for hemodialysis initiation depending on the stage of CKD. Adherence to the RHP, above and beyond age, gender and risk factors, decreased in 41.0% the probability of initiation of hemodialysis in patients with low risk of progression stages (CKD 1-3a). Age behaved as an independent risk factor in the high-risk stages, tripling even the probability of starting hemodialysis in the most gerontological patients. The mere fact of being male was a protective factor in all stages of CKD, decreasing by 49.0% (in high-risk stages) and 41.0% (in low-risk stages) the probability of starting hemodialysis. (Table 2)

**Table 2:** Effect of adherence to the renal health program (RHP) for starting dialysis according to stage of chronic renal disease, Health network Rebagliati-Lima Peru, 2013–2017.

	High Risk (Stage 3a-A3,3b,4)			Low Risk (Stage 1,2,3a A1 and A2)		
	HR Adjusted*	95%CI	p	HR Adjusted*	95%CI	p
Adherence to the RHP	1.17	0.84–1.63	0.361	0.59	0.41–0.85	0.005
Age in tertiles						
Tertile 1 [18–68]	Ref.			Ref.		
Tertile 2 [69–79]	2.09	1.51–2.90	< 0.001	1.08	0.73–1.59	0.697
Tertile 3 [80–103]	3.22	2.25–4.59	< 0.001	1.56	0.95–2.56	0.082
Gender						
Women	1			1		
Men	0.51	0.38–0.67	< 0.001	0.59	0.41–0.86	0.006
Risk Factors						
Others	1			1		
Hypertension	2.09	1.46–2.97	< 0.001	1.77	1.01–3.12	0.047
Diabetes	1.19	0.71–2.0	0.510	1.16	0.66–2.07	0.604
Diabetes and HBP	0.89	0.64–1.24	0.493	0.80	0.49–1.30	0.362

* Adjusted for gender, categorized age, CKD stage and risk factors.

CKD: chronic kidney disease; HBP: high blood pressure

Adherence to the RHP reduced in 13.0% the probability of dying independently of the other variables evaluated. Age increased the risk of mortality by almost five times in older ages. High-risk stages had more than twice the probability of dying than low-risk stages, and men had 42.0% more risk than women (data not shown). However, when assessing adherence behavior in each of the CKD strata, it has a significant effect only on the high-risk group, decreasing the probability of dying by 31.0% regardless of age, gender, and risk factors (Table 3). The risk strata of CKD were a modifying effect on the association between adherence to the RHP and mortality due to all causes.

**Table 3:** Effect of adherence to the renal health program (RHP) on mortality according to stage of chronic renal disease, Health network Rebagliati-Lima Peru, 2013–2017.

	High Risk (Stage 3a-A3,3b,4)			Low Risk (Stage 1,2,3a A1 and A2)		
	HR Adjusted*	95%CI	p	HR Adjusted*	95%CI	p
Adherence to the RHP	0.69	0.57–0.83	< 0.001	1.01	0.88–1.14	0.936
Age in tertiles						
Tertile 1 [18–68]	1			1		
Tertile 2 [69–79]	1.80	1.39–2.32	< 0.001	2.46	2.01–3.02	< 0.001
Tertile 3 [80–103]	3.21	2.53–4.07	< 0.001	5.93	4.89–7.19	< 0.001
Gender						
Women	1			1		
Men	1.35	1.18–1.56	< 0.001	1.46	1.30–1.63	< 0.001
Risk Factors						
Others	1			1		
Hypertension	0.56	0.47–0.65	< 0.001	0.63	0.54–0.74	< 0.001
Diabetes	0.71	0.52–0.97	0.030	0.85	0.69–1.04	0.113
Diabetes and HBP	0.85	0.70–1.02	0.084	0.72	0.61–0.86	< 0.001

* Adjusted for gender, categorized age, CKD stage and risk factors.

CKD: chronic kidney disease; HBP: high blood pressure

## DISCUSSION

This study describes the effectiveness of a secondary prevention strategy developed with basis on clinical guidelines, executed by a multidisciplinary team, with the participation of primary care and nephrology professionals to reduce mortality and progression to hemodialysis. Adherence to the guidelines changes the possibility of admission to dialysis in patients at low risk of progression, and significantly reduces all cause-mortality in patients at high risk of progression. Despite the increasing prevalence of CKD, there is little published evidence about the effectiveness of different models of care in improving outcomes in clinical practice^9^. Additionally, there is limited evidence that multidisciplinary models of care that provide care with a structured protocol have the potential to improve adherence and treatment goals.

Age is an independent risk factor, directly associated with higher risk of dialysis initiation in the high-risk CKD group. Tonelli et al.^17^ found that the probability of death was 145 times greater than the need for admission to RRT in patients over 80 years, due to the prevalence of multiple comorbidities, especially cardiovascular disease. Likewise, Soucie et al.^18^ state that age over 75 years is independently associated with a five-fold increase in the risk of death within 90 days after starting dialysis^19^. This early mortality is associated with predialysis health conditions. However, Hallan et al.^20^, in the meta-analysis to assess the possible interaction of age with clinical risk, conclude that low eGFR and high albuminuria are independently associated with mortality and dialysis admission.

The clinical risk parameters become essential in the evaluation and allow an early referral decision to the specialist. Baek SH et al.^21^ associated timely referral to the nephrologist with a 58.0% decrease in mortality in the first 90 days of dialysis initiation in elderly patients with chronic end-stage renal disease. Our findings showed that older patients are more likely to dialysis initiating, especially in high-risk CKD patients, in contrast to the findings of Tonelli et al.^17^ Similarly, Lundstrom et al.^22^ showed that the 5-year probability of admission to RRT is lower among patients over 75 years old due to the competitive risk with mortality.

Male gender becomes a protective factor in all stages of CKD, decreasing the probability of dialysis admission by almost half in this study. However, Marks et al.^23^ reported high rates of progression and initiation of RRT in males, suggesting that there is a biological component involved. While, Go et al.^24^, in assessing the risk of rapid progression for CKD among more than 36,000 American patients, found no significant difference for men and women. We propose that in addition to biological variables, cultural and social variables may be involved. In our environment, male patients are cared for by their families (daughters and wives), who ensure that they adhere to their treatment and diet.

The effectiveness of a secondary prevention strategy, using a multidisciplinary team, had already been observed in the economic evaluation of CanPREVENT (The Canadian Prevention of Renal and Cardiovascular Endpoints Trial). It concludes that the use of a team (nurse, nephrologist) in the consultation uses fewer resources and leads to lower health care costs without reducing the quality of life for patients with CKD.^25^ In addition, the Korean national P4P (pre-dialysis program), implemented with a multidisciplinary team care model, significantly improved the quality of CKD management, and reduced the risk of early mortality and health care expenses.^26^ Finally, Strand et al.^27^, in their systematic review in 2012, established that multidisciplinary care is effective in delaying the progression of CKD in adults, especially in the advanced stages. Education becomes an important component of care by increasing knowledge and understanding of the causes of CKD. Our work emphasizes the importance of teamwork to achieve specific goals, which would be achieved even in patients in advanced stages of CKD.

The adherence to the RHP was 23.6%. This is a low percentage in relation to the average number of patients adhering to a treatment scheme for chronic diseases, which can reach 74.0% in diabetic patients^10^. However, a study of CKD patients in the Netherlands showed that adherence to treatment reached 54.0%, but decreased as the stage of disease was more severe^28^. In our case, it may be due to problems in the health system, lack of appointments, bureaucracy, but may also reflect under-registration in the computer application, or the fact that we established a minimum number of evaluations per year according to stage to consider adherence.

Our study showed that adherence is strongly influenced by the stage of disease. Two meta-analyses^29^ reported that low-risk CKD patients who adhere to RHP are less likely to begin dialysis. In addition, they conclude that low eGFR and high albuminuria should be considered at least as relevant a risk factor for CKD progression to dialysis in diabetics or hypertensive patients. Likewise, in clinical evaluation, models combining albuminuria and eGFR should be considered since they add significant predictive information on the risk of admission to RRT or death compared to models containing only one of the parameters in an isolated form^30^.

The silent progression of CKD, its association with cardiovascular disease and the high cost of treatment make this disease a major public health concern, especially since kidney replacement therapy is out of financial reach for most Peruvians under their current health system^2^. Despite this, the country still has an incipient renal prevention program, having achieved partial development in social security assistance by the National Renal Health Plan approved in 2008, whose objectives are: to decrease the incidence of CKD in the population at risk, to organize health care, to strengthen the capacity of primary care resolution and to establish a surveillance system for renal health. This work describes the first experience in secondary prevention by levels of care, with computerized registration in this health system.

A limitation of our study is that only data from recording information from patients in monitoring and not clinical records were used. An immediate effect is the difficulty in managing the databases because they come from different sources, and the consequent greater probability of finding missing values in some variables. However, given the sample size after methodological exclusions (n = 20,354), the proportion of missing values (4.7%) does not represent a serious threat to the validity of the results. On the other hand, there is less flexibility to model the categorization of variables, since the criteria that define them come from the very design of the records. An example is the category “others” in the risk factors including people aged over 55. Although age is a variable in itself, it is not possible to exclude those over 55 and put them in a new category, since it is impossible to differentiate patients whose risk factor is only their age from those with more than one reason for being in “others” category. We also use an expert opinion definition of adherence to establish the study groups. Although this is a valid method, because we are identifying the intervention group retrospectively, and based on a rule that is closely related to the patient’s inclination to participate in the intervention, there is a probability of selection bias that could lead us to overestimate the effect of the intervention. In addition, our study cohort only measured adherence in the first year, due to the difficulty in establishing criteria over time, since CKD is a dynamic disease, in which each patient can be stable, in progress or in remission. The average monitoring time of two years makes it difficult to assess survival because it is a relatively short period, so the findings should be interpreted with caution.

Adherence to the renal health program has differentiated effects for hemodialysis initiating depending on the stage of CKD. It is effective in reducing the initiation of renal replacement therapy when patients are identified in early stages and has a significant effect on mortality in the group at high risk of progression, regardless of other factors such as age or gender.
