# Supplementary material for: Effectiveness of adherence to a renal health program in a health network in Peru
Source: Rev Saude Publica. 2020 Aug 10;54:80. doi: 10.11606/s1518-8787.2020054002109 (PMC7416761; doi:10.11606/s1518-8787.2020054002109)
Supplement: Translation

# Translation: Efectividad de la adherencia a un programa de salud renal en una red sanitaria de Perú

**Authors:** Jessica Bravo-Zúñiga, Enrique M. Saldarriaga, Ricardo Chávez-Gómez, Jungmei Gálvez-Inga, Renzo Valdivia-Vega, Mirko Villavicencio-Carranza, José Espejo-Sotelo, Carola Medina-Sal y Rosas, Víctor Suarez-Moreno, Yamilee Hurtado-Roca

**Affiliations:** I Perú Unidad de Salud Renal, Departamento de Nefrología, Hospital Nacional Edgardo Rebagliati Martins, EsSalud. Lima, Perú; II Perú Instituto de Evaluación de Tecnologías en Salud e Investigación (IETSI), EsSalud. Lima, Perú; III USA Policy and Economics (CHOICE) Institute. The Comparative Health Outcomes. Seattle, Washington, USA; IV Perú Sin Brechas S.A.C.

**Keywords:** Insuficiencia Renal Crónica, prevención & control, Prestación Integrada de Atención de Salud, Comunicación Interdisciplinaria, Evaluación de Eficacia-Efectividad de Intervenciones

## Abstract

**OBJETIVO:**

Evaluar la efectividad de la adherencia a un programa de salud renal en la reducción de mortalidad y progresión a hemodiálisis.

**MÉTODOS:**

Utilizamos una base de datos que condensaba el seguimiento de los pacientes (2013-2017), los ingresos a diálisis de los mismos y la mortalidad por todas las causas en Perú. La adherencia al programa se estableció con el cumplimiento de visitas mínimas durante su primer año de seguimiento. La efectividad de la adherencia al programa se midió en términos de debut a hemodiálisis o muerte por todas las causas. Se utilizaron curvas de Kaplan-Meier, test de diferencias en la distribución (Log-Rank test) y métodos de análisis de supervivencia. Los análisis se realizaron utilizando R estudio 3.5.0

**RESULTADOS:**

Fueron evaluados 20.354 participantes, 54,1% varones, edad media de 72,1 años, con un seguimiento medio de 2,2 años; 15.279 (75.1%) tuvieron ERC en estadios tempranos (estadio 1 al 3a). La adherencia disminuyó en un 41,0% el riesgo de terapia de reemplazo renal (HR = 0,59; IC95% 0,41–0,85) en el grupo de bajo riesgo y en un 31,0% (HR = 0,69; IC95% 0,57–0,83) la mortalidad en el grupo de alto riesgo.

**CONCLUSIONES:**

La estrategia de cuidado multidisciplinario con evaluaciones estandarizadas según estadio es efectiva en reducir el ingreso a terapia de reemplazo renal cuando se identifica al paciente en estadios tempranos y en reducir la mortalidad en estadios avanzados.

## INTRODUCCIÓN

La prevalencia global de enfermedad renal crónica (ERC) para todas las etapas de la vida se ha estimado entre, 11,0 y 13,0%. La prevalencia de ERC en el Perú es del 13,2%^1^. Sin embargo, en la capital, Lima, donde vive un tercio de la población, se ha estimado en 20,7%^2^. La enfermedad renal es la sexta causa de muerte en el país, con aumento de un 28,0% en los últimos 10 años. Las ciudades que muestran mayor crecimiento en la proporción de muertes por ERC están ubicadas en la región de la sierra, donde hay pocos nefrólogos^3^, condicionando una referencia tardía al especialista^4^.

La progresión natural de la ERC lleva a los pacientes en sus estadios finales al uso de terapia de reemplazo renal (TRR). La cantidad de pacientes en diálisis peritoneal o hemodiálisis es de 415 pacientes por millón de habitantes (pmh) en el Perú^5^, proporción baja en comparación a la media para Latinoamérica (660 pmh)^6^. Es decir, tenemos 50,0% de pacientes con ERC en estadio final que no accede a TRR en relación a otros países de la región. Más aún, el Ministerio de Salud reconoce que la demanda ha superado ampliamente la oferta nacional^5^.

El impacto económico de la ERC es global, llega a todos los países, independientemente del nivel de desarrollo y el modelo de sanidad. El promedio de gasto por año en TRR puede llegar a 100 mil dólares, mientras que la inversión en cuidados para enlentecer la ERC llega a 10 mil dólares en promedio. Por ello, estudios advierten de la importancia de la prevención primaria y secundaria de la ERC como política de asistencia sanitaria^7^.

El 30% de los peruanos cuenta con seguridad social, y es en este sistema sanitario donde la diálisis genera una importante carga financiera. En el 2017, el gasto en diálisis superó los 1,2 millones de soles, es decir, el 6% del presupuesto institucional^8^. Adicionalmente, someterse a TRR tiene un impacto negativo importante en la calidad de vida de los pacientes. Reducir el número de pacientes nuevos que requieran TRR es un escenario muy deseable a nivel individual y social.

Se requieren estrategias de cuidado de la ERC para retardar su evolución a diálisis o muerte. Las más exitosas incluyen programas con cuidado multidisciplinario, con control de la enfermedad de fondo y tratamiento farmacológico para el control de los factores de riesgo modificables. El tiempo de seguimiento y la adherencia al programa son los aspectos que pueden hacer la diferencia^9^.

La falta de adherencia a la terapia farmacológica crónica aumenta significativamente la carga de enfermedad. Hay factores asociados a esta pobre adherencia como el costo, la complejidad y efectos adversos de la medicación; el seguimiento inadecuado, la relación deficiente entre el paciente y el médico, y las barreras para acceder a los centros de salud, circunstancias más frecuentes en las economías en desarrollo^10^.

La Organización Mundial de la Salud (OMS) promueve tácticas que apuntan a un manejo oportuno de la enfermedad y comorbilidades asociadas, como la diabetes y la hipertensión arterial (HTA)^11^. Inmerso en este contexto nacional y considerando las recomendaciones de la OMS, la Unidad de Salud Renal del Hospital Nacional Edgardo Rebagliati, de la Seguridad Social de Perú (EsSalud), comenzó la implementación de un plan de prevención secundaria en 2013. Esta intervención se centra en un manejo multidisciplinario de pacientes con factores de riesgo para la ERC como diabetes, hipertensión y edad mayor de 55 años, con la finalidad de reducir la progresión de ERC, controlar las enfermedades de fondo y reducir la mortalidad general^12^. El objetivo de este estudio fue evaluar la efectividad de la adherencia a un programa de salud renal (PSR) en la reducción de mortalidad y progresión a hemodiálisis.

## MÉTODOS

Análisis de los datos de seguimiento de una cohorte de pacientes conformada por aquellos que ingresaron al programa de salud renal de la Red Sanitaria Rebagliati, Lima-Perú, desde el 01 de enero del 2013 hasta el 31 de diciembre del 2017. El seguimiento finalizó el 31 de julio del 2018.

Fueron utilizadas tres bases de datos: 1) De seguimiento de los pacientes incluidos en la PSR de la Red sanitaria Rebagliati entre 2013 y 2017, 2) De hemodiálisis de la Red Sanitaria Rebagliati, de donde fueron extraídas las fechas de inicio de hemodiálisis de los pacientes incluidos y 3) De mortalidad, construida a partir de los datos (hasta julio del 2018) del registro nacional de identificación y estado civil del Perú. Los tres archivos fueron proporcionados con un código alfanumérico de identificación único (CI) para cada paciente que garantizó el anonimato de la información. Con estas tres bases de datos, se diseñó una única identificada por el CI que incluía 23.144 sujetos. Se excluyeron datos de sujetos que no calificaban como ERC (n = 1.429), ERC estadio 5 (n = 393), menores de 18 años (n = 19), fuera del periodo de tamizaje (n = 5), datos inconsistentes (n = 125), datos faltantes (estadio ERC = 424 y fecha de fallecimiento = 542).

La variable de exposición estuvo determinada por la adherencia al programa de salud renal con cuidado multidisciplinario (médico, enfermera, nutricionista). Ésta se estableció por recomendación de expertos de la Unidad de Salud Renal y consenso de los investigadores a partir del cumplimiento de visitas durante su primer año de ingreso: para los pacientes con ERC estadio 1 y 2 = mínimo una visita, para ERC estadio 3a y 3b = mínimo dos visitas y para ERC estadio 4 = mínimo tres visitas. Los estadios de ERC fueron categorizados en función a su riesgo de progresión (tomando en cuenta la TFG y albuminuria) como ERC con bajo riesgo de progresión: Estadio 1 (Tasa de filtración glomerular estimada [TFGe] > 90ml/min y Relación albumina creatinina [RAC) > 30mg/g], Estadio 2 (90 < TFGe < 60 ml/min y RAC > 30mg/g), y Estadio 3a (60 < TFGe < 45 ml/min y ACR < 300mg/g); y ERC con alto riesgo de progresión: Estadio 3a (60 < TFGe < 45 ml/min y ACR > 300mg/g), Estadio 3b (45 < TFGe < 30ml/min), y Estadio 4 (15 < TFGe < 30ml/min), de acuerdo con la guía KDIGO^13^. Los factores de riesgo incluyeron hipertensión, diabetes mellitus y otros (en esta categoría se incluyeron: litiasis renal, glomerulopatías primarias y secundarias, uropatías obstructivas, y edad mayor de 55 años).

El desenlace del análisis estuvo definido por tiempo a evento hemodiálisis o muerte (desde el ingreso a la cohorte hasta la presentación de cualquiera de los dos sucesos, el que ocurriese primero). La efectividad de la adherencia al programa se midió en términos de la presencia de estos desenlaces.

Para el análisis de supervivencia se utilizaron curvas de Kaplan-Meier y se aplicó un test de diferencias en la distribución (Log-Rank test) para determinar si las curvas fueron estadísticamente diferentes entre sí. Se utilizaron modelos de supervivencia para evaluar la asociación entre la adherencia al PSR y presentar hemodiálisis o morir. Esta asociación fue estimada de manera cruda y ajustada por las covariables identificadas como potenciales factores de confusión en la asociación principal (sexo, edad categorizada en terciles, factores de riesgo y estadios de ERC). En el caso de la progresión a hemodiálisis, se realizó un análisis de supervivencia por riesgo competitivo, mediante el cálculo, a partir de una regresión paramétrica, de curvas de incidencia acumuladas específicas para cada riesgo^14^. Se utilizó el paquete “Cause-specific competing-risk survival analysis” de R estudio para la estimación^15^. Fueron utilizadas regresiones de Cox para Hazards proporcionales en el caso de la muerte como evento. En ambos casos, se obtuvieron ratios de Hazard (riesgo instantáneo), con un nivel del 95% de confianza y p < 0,05. Los análisis se realizaron utilizando el paquete estadístico R estudio 3.5.0^16^.

Este estudio fue aprobado por el Comité de Investigación y Ética Institucional del Hospital Edgardo Rebagliati Martins.

## RESULTADOS

Se evaluaron 20.354 participantes. De estos, el 54,3% fueron varones y tuvieron una edad promedio de 72,9 años [desviación estándar (DS = 12,6)], mayoritariamente con ERC en estadio 3a (41,4%) y con hipertensión arterial como el factor de riesgo (38,7%) más frecuente. El tiempo promedio de seguimiento fue de 2,2 años (DS = 1,4), presentándose 1,7% casos de debut en hemodiálisis y 10,9% muertes por todas las causas durante el periodo de seguimiento. Del total de la población evaluada, 23,6% cumplieron con criterios para considerarse adherentes al programa de salud renal y 75,1% fueron identificados en el grupo de bajo riesgo de progresión (Tabla 1).

**Tabla 1:** Características de los pacientes del programa de salud renal (PSR), según adherencia, Red sanitaria Rebagliati-Lima Perú, 2013–2017.

Característica	Total		Adherencia al PSR		No adherencia al PSR	
	n	%	n	%	n	%
	20.354		4.801	23,6	15.553	76,4
Tiempo de seguimiento (años)*	2,2 (1,4)		2,4 (1,3)		2,1 (1,4)	
Edad de ingreso (años)*	72,9 (12,6)		72,6 (12,3)		73,1 (12,7)	
Edad en terciles						
Tercil 1 [18–68]	6.514	32,0	1.558	32,5	4.956	31,9
Tercil 2 [69–79]	6.993	34,4	1.747	36,4	5.246	33,7
Tercil 3 [80–103]	6.847	33,6	1.496	31,2	5.351	34,4
Estadio inicial de la ERC						
1	3.614	17,8	1.053	21,9	2.561	16,5
2	4.999	24,6	1.683	35,1	3.316	21,3
3a	8.433	41,4	1.376	28,7	7.057	45,4
3b	2.207	10,8	531	11,1	1.676	10,8
4	1.101	5,4	158	3,3	943	6,1
Estadio inicial de ERC categorizado						
Bajo riesgo ERC	15.279	75,1	3.687	76,8	11.592	74,5
Alto riesgo ERC	3.708	18,2	795	16,6	2.913	18,7
Sexo						
Mujeres	9.294	45,7	2.438	50,8	6.856	44,1
Hombres	11.06	54,3	2.363	49,2	8.697	55,9
Factores de riesgo						
Otros	5.347	26,3	1.012	21,1	4.335	27,9
Hipertensión	7.877	38,7	1.763	36,7	6.114	39,3
Diabetes	2.795	13,7	765	15,9	2.03	13,1
Diabetes e HTA	4.335	21,3	1.261	26,3	3.074	19,8
Total de eventos (debut en diálisis)	347	1,7	92	1,9	255	1,6
Total de fallecimientos	2.224	10,9	491	10,2	1.733	11,1

* Media (desviación estándar)

ERC: enfermedad renal crónica; HTA: hipertensión arterial

Las curvas de Kaplan-Meier para ambos grupos con el inicio de diálisis como el evento de interés mostraron una proporción de personas sin diálisis superior al 95% al final de los cinco años de seguimiento (Figura 1). Estas proporciones fueron consistentes con el pequeño número de personas que pasan a TRR (Tabla 1). La supervivencia a la diálisis fue menor en el grupo adherido al PSR (95,8%; IC95% 94,3–97,3) que en el control (96,5; IC95% 95,9–97,1). Sin embargo, al aplicar el Long-Rank test se evidenció que las curvas no fueron significativamente diferentes entre sí (valor de p = 0,093); por lo tanto, la adherencia al PSR no modificó el ingreso a TRR.

**Figura 1:** Curvas de Kaplan-Meier para evaluar progresión a hemodiálisis en pacientes del programa de salud renal según adherencia, Red sanitaria Rebagliati-Lima Perú, 2013–2017.

En las curvas de mortalidad global de Kaplan-Meier para ambos grupos, el grupo no adherido al PSR tuvo una menor supervivencia que el grupo adherido en todo el período de estudio (Figura 2). Al final de los cinco años siguientes, la supervivencia fue de 70,9% (IC95% 65,8–76,8) en el grupo adherido y 61,5% (IC95% 55,1–68,6) en el grupo no adherido. La prueba Long-Rank mostró que las curvas fueron estadísticamente diferentes entre sí (p < 0,001), por lo que adherirse al PSR modificaría la mortalidad.

**Figura 2:** Curva de Kaplan-Meier para evaluar mortalidad en pacientes del programa de salud renal, según adherencia, Red sanitaria Rebagliati-Lima Perú, 2013–2017.

Para evaluar el efecto de la adherencia al PSR en el debut a hemodiálisis, se realizaron análisis de regresión crudos y ajustados, evidenciando un cambio en la direccionalidad de la asociación. Se identificó que el estadio de la ERC estaría modificando el efecto en esta asociación (datos no mostrados). Así, los resultados indicaron que la adherencia al PSR tuvo efectos diferenciados para el debut a hemodiálisis dependiendo del estadio de la ERC. De tal forma, la adherencia al PSR, independientemente de la edad, sexo y factores de riesgo, disminuyó en un 41,0% la probabilidad de debutar a hemodiálisis en los pacientes con estadios de bajo riesgo de progresión (ERC 1-3a). La edad se comportó como un factor de riesgo independiente en los estadios de alto riesgo, triplicando incluso la probabilidad de debutar en hemodiálisis en los pacientes más gerontes. El solo hecho de ser varón fue un factor protector en todos los estadios de la ERC, disminuyendo en 49,0% (en estadios de alto riesgo) y en 41,0% (en estadios de bajo riesgo) la probabilidad de debutar a hemodiálisis (Tabla 2).

**Tabla 2:** Efecto de la adherencia al programa de salud renal (PSR) para debutar en diálisis según estadio de la enfermedad renal crónica, Red sanitaria Rebagliati-Lima Perú, 2013–2017.

	Alto riesgo (Estadio 3a-A3,3b,4)			Bajo riesgo (Estadio 1,2,3a A1 y A2)		
	HR ajustado*	IC95%	p	HR ajustado*	IC95%	p
Adherencia al PSR	1,17	0.84–1,63	0,361	0,59	0,41–0,85	0,005
Edad en terciles						
Tercil 1 [18–68]	Ref.			Ref.		
Tercil 2 [69–79]	2,09	1,51–2,90	< 0,001	1,08	0,73–1,59	0,697
Tercil 3 [80–103]	3,22	2,25–4,59	< 0,001	1,56	0,95–2,56	0,082
Sexo						
Mujeres	1			1		
Hombres	0,51	0,38–0,67	< 0,001	0,59	0,41–0,86	0,006
Factores de riesgo						
Otros	1			1		
Hipertensión	2,09	1,46–2,97	< 0,001	1,77	1,01–3,12	0,047
Diabetes	1,19	0,71–2,0	0,510	1,16	0,66–2,07	0,604
Diabetes e HTA	0,89	0,64–1,24	0,493	0,80	0,49–1,30	0,362

* Ajustado por sexo, edad categorizada, estadio de ERC y factores de riesgo.

ERC: enfermedad renal crónica; HTA: hipertensión arterial

La adherencia al PSR disminuyó en un 13,0% la probabilidad de morir independientemente de las demás variables evaluadas. La edad incrementó el riesgo de mortalidad en casi cinco veces en las edades más avanzadas. Los estadios de alto riesgo tuvieron más del doble de probabilidad de morir respecto a los de bajo riesgo y los hombres tuvieron 42,0% más riesgo que las mujeres (datos no mostrados). Sin embargo, al evaluar el comportamiento de la adherencia en cada uno de los estratos de ERC, tiene un efecto significativo sólo en el grupo de alto riesgo, disminuyendo en un 31,0% la probabilidad de morir independientemente de la edad, el sexo y los factores de riesgo (Tabla 3). Los estratos de riesgo de la ERC fueron un efecto modificador de la asociación entre la adherencia al PSR y mortalidad por todas las causas.

**Tabla 3:** Efecto de la adherencia al programa de salud renal (PSR) en la mortalidad según estadio de la enfermedad renal crónica, Red sanitaria Rebagliati-Lima Perú, 2013–2017.

	Alto riesgo (Estadio 3a-A3,3b,4)			Bajo riesgo (Estadio 1,2, 3a A1 y A2)		
	HR ajustado*	IC95%	p	HR ajustado*	IC95%	p
Adherencia al PSR	0,69	0,57–0,83	< 0,001	1,01	0,88–1,14	0,936
Edad en terciles						
Tercil 1 [18–68]	1			1		
Tercil 2 [69–79]	1,80	1,39–2,32	< 0,001	2,46	2,01–3,02	< 0,001
Tercil 3 [80–103]	3,21	2,53–4,07	< 0,001	5,93	4,89–7,19	< 0,001
Sexo						
Mujeres	1			1		
Hombres	1,35	1,18–1,56	< 0,001	1,46	1,30–1,63	< 0,001
Factores de riesgo						
Otros	1			1		
Hipertensión	0,56	0,47–0,65	< 0,001	0,63	0,54–0,74	< 0,001
Diabetes	0,71	0,52–0,97	0,030	0,85	0,69–1,04	0,113
Diabetes e HTA	0,85	0,70–1,02	0,084	0,72	0,61–0,86	< 0,001

*Ajustado por sexo, edad categorizada, estadio de ERC y factores de riesgo.

ERC: enfermedad renal crónica; HTA: hipertensión arterial

## DISCUSIÓN

Este estudio describe la efectividad de un programa de salud renal organizado con base en guías clínicas, el cuidado por un equipo multidisciplinario, y establecido por niveles de atención con la participación de profesionales de atención primaria y de nefrología en términos de reducción de mortalidad y de progresión a hemodiálisis. La adherencia al mismo modifica la posibilidad de ingresar a diálisis en los pacientes de bajo riesgo de progresión, y disminuye significativamente la mortalidad por todas las causas en los pacientes con alto riesgo de progresión. A pesar del incremento de la prevalencia de la ERC, hay poca evidencia publicada sobre la efectividad de los diferentes modelos de cuidado en mejorar los resultados en la práctica clínica^9^. Adicionalmente, hay evidencia limitada que los modelos de cuidado multidisciplinario que brindan atención con un protocolo estructurado pueden mejorar la adherencia y los objetivos del tratamiento.

La edad se comporta como un factor de riesgo independiente, evidenciando que a mayor edad mayor riesgo de mortalidad y mayor riesgo de debutar en diálisis; en el grupo con ERC de alto riesgo. Tonelli y col.^17^ encontraron que la probabilidad de muerte era 145 veces mayor que la necesidad de ingreso a TRR en pacientes mayores de 80 años, debido a la prevalencia de múltiples comorbilidades, sobre todo, enfermedad cardiovascular. Así mismo, Soucie y col.^18^ afirman que la edad mayor de 75 años se asocia de forma independiente con un incremento de cinco veces el riesgo de muerte dentro de los 90 días posteriores al inicio de la diálisis^19^. Esta mortalidad temprana está asociada a condiciones de salud previas al inicio de diálisis. Sin embargo, Hallan y col.^20^, en el metanálisis para evaluar la posible interacción de la edad con el riesgo clínico, concluyen que la TFGe baja y la albuminuria elevada se asocian de forma independiente con la mortalidad y el ingreso a diálisis.

Los parámetros clínicos de riesgo se vuelven imprescindibles en la evaluación y permiten una decisión de referencia temprana al especialista. Baek SH y col.^21^ asociaron la referencia oportuna al nefrólogo con una disminución de 58,0% en la mortalidad en los primeros 90 días de inicio de diálisis en pacientes ancianos con enfermedad renal crónica terminal. Nuestros hallazgos evidenciaron que a mayor edad los pacientes tienen mayor probabilidad de debutar en diálisis sobre todo en los pacientes con ERC de alto riesgo, en contraposición a los hallazgos de Tonelli et al.^17^ Del mismo modo, Lundstrom et al.^22^ evidenciaron que la probabilidad a cinco años de ingreso a TRR es más baja entre los pacientes mayores de 75 años debido al riesgo competitivo con mortalidad.

El sexo masculino se convierte en un factor protector en todos los estadios de ERC, disminuyendo en casi la mitad la probabilidad de ingreso a diálisis en este estudio. Sin embargo, Marks et al.^23^ reportaron altas tasas de progresión e inicio de TRR en varones, sugiriendo que hay un componente biológico involucrado. Mientras que, Go et al.^24^, al evaluar el riesgo de rápida progresión para ERC entre más de 36.000 pacientes estadounidenses, no encontraron diferencia significativa para varones y mujeres. Planteamos que pueden intervenir más que variables biológicas, variables culturales y sociales. En nuestro medio, los pacientes varones son cuidados por sus familiares (hijas y esposas), quienes se encargan de que cumplan su tratamiento y su dieta.

La prevención secundaria, utilizando un equipo multidisciplinario, ya había sido apoyada por la evaluación económica del CanPREVENT (The Canadian Prevention of Renal and Cardiovascular Endpoints Trial). Se concluye que el uso de un equipo (enfermera, nefrólogo) en la consulta utiliza menos recursos y conlleva menor costo para el cuidado de la salud sin reducir la calidad de vida para pacientes con ERC^25^. Así mismo, el programa P4P (programa de pre diálisis) nacional coreano, implementado con un modelo de atención en equipo multidisciplinario, mejoró significativamente la calidad del manejo de ERC, y redujo el riesgo de mortalidad temprana y los gastos de atención de la salud.^26^ Finalmente, Strand et al.^27^, en su revisión sistemática en el 2012, establecieron que la atención multidisciplinaria es eficaz para retrasar la progresión de la ERC en adultos, especialmente en los estadios avanzados. La educación se convierte en un componente importante de la atención logrando aumentar el conocimiento y la comprensión de las causas de la ERC. Nuestro trabajo incide en la importancia de trabajar en equipo para lograr objetivos específicos, que se conseguirían incluso en los pacientes con estadios avanzados.

La adherencia al programa de salud renal fue de 23,6%. Esta es una cifra baja en relación al promedio de adherencia a un esquema de tratamiento en enfermedades crónicas, que puede llegar a un 74,0% en pacientes diabéticos^10^. No obstante, un trabajo realizado en pacientes con ERC en Holanda mostró que la adherencia terapéutica llegaba al 54,0%, pero disminuía conforme el estadio de enfermedad era más severo^28^. En nuestro caso, esto puede deberse a problemas propios del sistema de salud, falta de citas, burocracia, pero también puede reflejar subregistro en el aplicativo informático, o el hecho de que establecimos un número de evaluaciones mínimas por año según estadio para considerar adherencia.

Nuestro estudio evidenció que la adherencia está fuertemente influenciada por el estadio de enfermedad. Dos metanálisis^29^ reportaron que los pacientes con ERC de bajo riesgo que se adhieren al PSR tienen menor probabilidad de debutar en diálisis. Adicionalmente, concluyen que la TFGe baja y la albuminuria elevada se deben considerar al menos como un factor de riesgo igualmente relevante para la evolución a ERC en diálisis en personas diabéticas o hipertensas. Así mismo, en la evaluación clínica, se debe considerar modelos que combinen albuminuria y TFGe pues agregan información predictiva significativa sobre el riesgo de ingreso a TRR o muerte en comparación con los modelos que solo contienen uno de los parámetros en forma aislada^30^.

La progresión silenciosa de la ERC, su asociación con enfermedad cardiovascular y el costo elevado del tratamiento hacen que esta enfermedad sea de gran preocupación para la salud pública, sobre todo, porque la terapia de reemplazo renal está fuera del alcance financiero para la mayoría de peruanos bajo su actual sistema de salud^2^. A pesar de ello, el país aún tiene un programa de prevención renal incipiente, habiendo logrado un desarrollo parcial en la seguridad social a través del Plan Nacional de Salud Renal aprobado en 2008, cuyos objetivos son: disminuir la incidencia de la ERC en la población de riesgo, organizar la atención de salud, fortalecer la capacidad de resolución de atención primaria y establecer un sistema de vigilancia para la salud renal. Este trabajo describe la primera experiencia en prevención secundaria por niveles de atención, con registro informático en este sistema de salud.

Una limitación de nuestro estudio es que solo se utilizaron los datos de registro de información de pacientes en seguimiento y no registros clínicos. Un efecto inmediato es la dificultad en el manejo de las bases de datos por provenir de diversas fuentes, y la consecuente mayor probabilidad de encontrar valores perdidos en algunas variables. No obstante, dado el tamaño de muestra luego de exclusiones metodológicas (n = 20.354), la proporción de valores perdidos (4,7%) no representa una amenaza seria a la validez de los resultados. Por otro lado, se tiene menos flexibilidad para modelar la categorización de las variables, puesto que los criterios que las definen provienen del diseño mismo de los registros. Un ejemplo es la categoría “otros” en los factores de riesgo que incluye personas mayores de 55 años. Aunque la edad es una variable en sí misma, no es posible excluir a los mayores de 55 y ponerlos en una nueva categoría, ya que es imposible diferenciar los pacientes cuyo factor de riesgo es únicamente su edad, de aquellos con más de una razón para estar en “otros”. Asimismo, usamos una definición de adherencia con base en la opinión de expertos para establecer los grupos de estudio. Aunque este es un método válido, debido a que estamos identificando retrospectivamente el grupo de intervención, y basándonos en una regla muy relacionada con la disposición del paciente a participar en la intervención, existe una probabilidad de sesgo de selección que podría llevarnos a sobreestimar el efecto de la intervención. Además, nuestra cohorte de estudio solo midió la adherencia en el primer año, debido a la dificultad para establecer los criterios en el tiempo, ya que la ERC es una enfermedad dinámica, donde cada paciente puede mantenerse estable, progresar o remitir. El tiempo de seguimiento promedio de dos años dificulta valorar la supervivencia por ser un periodo relativamente corto, por lo cual los hallazgos deben ser interpretados con cautela.

La adherencia al programa de salud renal tiene efectos diferenciados para el debut en hemodiálisis dependiendo del estadio de la ERC. Es efectiva en reducir el inicio de terapia de reemplazo renal cuando se identifican pacientes en estadios tempranos y tiene un efecto significativo sobre la mortalidad en el grupo de alto riesgo de progresión, independientemente de otros factores como la edad o el género.
